# Balancing Yields and Sustainability: An Eco-Friendly Approach to Losartan Synthesis Using Green Palladium Nanoparticles

**DOI:** 10.3390/molecules30112314

**Published:** 2025-05-25

**Authors:** Edith M. Antunes, Yusuf A. Adegoke, Sinazo Mgwigwi, John J. Bolton, Sarel F. Malan, Denzil R. Beukes

**Affiliations:** 1 Chemistry Department, University of the Western Cape, Private Bag X17, Bellville, Cape Town 7535, South Africa; ebeukes@uwc.ac.za; 2 School of Pharmacy, University of the Western Cape, Private Bag X17, Bellville, Cape Town 7535, South Africa; adegoke.adeyemi@gmail.com (Y.A.A.); 3761240@myuwc.ac.za (S.M.); sfmalan@uwc.ac.za (S.F.M.); 3 Department of Biological Sciences, University of Cape Town, Rondebosch, Cape Town 7701, South Africa; john.bolton@uct.ac.za

**Keywords:** green chemistry, losartan synthesis, palladium nanoparticles, Suzuki–Miyaura coupling, *Sargassum incisifolium*

## Abstract

This study presents a sustainable, environmentally friendly synthetic route for the production of key intermediates in losartan using palladium nanoparticles (PdNPs) derived from a brown seaweed, *Sargassum incisifolium*, as a recyclable nanocatalyst. A key intermediate, biaryl, was synthesized with an excellent yield (98%) via Suzuki–Miyaura coupling between 2-bromobenzonitrile and 4-methylphenylboronic acid, catalyzed using bio-derived PdNPs under mild conditions. Subsequent bromination using *N*-bromosuccinimide (NBS) under LED light, followed by imidazole coupling and tetrazole ring formation, allowed for the production of losartan with an overall yield of 27%. The PdNP catalyst exhibited high stability and recyclability, as well as strong catalytic activity, even at lower loadings, and nitrosamine formation was not detected. While the overall yield was lower than that of traditional industrial methods, this was due to the deliberate avoidance of the use of toxic reagents, hazardous solvents, and protection/deprotection steps commonly used in conventional routes. This trade-off marks a shift in pharmaceutical process development, where environmental and safety considerations are increasingly prioritized in line with green chemistry and regulatory frameworks. This study provides a foundation for green scaling up strategies, incorporating sustainability principles into drug synthesis.

## 1. Introduction

Cardiovascular regulation and blood pressure maintenance are facilitated by the renin–angiotensin system (RAS), where the stimulation of specific receptors in various organs is enabled by the principal active hormone angiotensin II [1,2,3]. Angiotensin II induces vasoconstriction, resulting in an increase in blood pressure, making the interruption of the RAS an effective approach for controlling hypertension. Consequently, angiotensin-converting enzyme (ACE) inhibitors have been successfully used in managing hypertension, anxiety, glaucoma, and cardiac events [4]. Angiotensin receptors are classified as either AT_1_ and AT_2_, with most angiotensin II activity, including vasoconstriction, aldosterone release, renal sodium reabsorption, and vasopressin secretion, mediated by AT_1_ receptors [5,6].

Angiotensin II receptor (AT_1_R) antagonists, such as losartan, are important in managing hypertension [7,8]. Losartan became the first nonpeptide AT_1_R approved by the U.S. Food and Drug Administration for clinical use in hypertension alone or in combination with other antihypertensive agents [9]. Despite its therapeutic importance, existing methods for producing losartan are often hindered by low yields [10], labour-intensive workup procedures [11,12], and the use of toxic and expensive reagents such as organotin reagents [13], which complicates recovery and disposal.

**Scheme 1:**
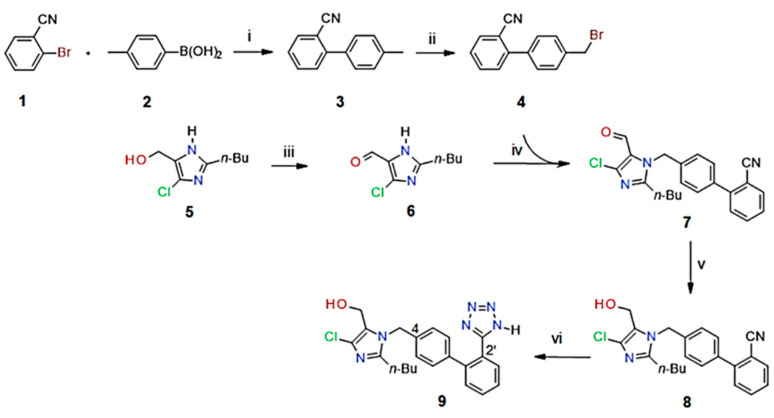
Synthesis of losartan (**9**). Conditions: (i) PdNPs, K_2_CO_3_, acetone/H_2_O (1:1), 35 °C, 24 h; (ii) NBS, *hν*, ACN, 70 °C, 2 h; (iii) TEMPO, iodine, toluene, aq. KHCO_3_, 20 °C; (iv) K_2_CO_3_, EtOH, 45 °C, 24 h; (v) NaBH_4_, MeOH; (vi) NaN_3_, ZnBr_2_, n-BuOH, 100 °C, 48 h.

The biphenyl scaffold is a key structural feature of most AT_1_R antagonists, including losartan (**9**, Scheme 1). In losartan, this scaffold incorporates a heterocyclic group at the 4-position and a carboxylic acid or tetrazole group at the 2′-position [10]. Forming a biphenyl moiety through aryl–aryl coupling is a critical step in the synthesis of these compounds. Conventional approaches include Ullman coupling, the nucleophilic aromatic substitution of activated benzoic acid derivatives, the Ni-catalyzed coupling of 4-methylphenyl magnesium bromide with 2-bromobenzonitrile, and Suzuki–Miyaura coupling between aryl halides and aryl boronic acids [14]. In addition to biphenyl formation, the synthesis of losartan involves the introduction of a tetrazole moiety through a [2 + 3] cycloaddition of azides and nitriles [15,16]. While conventional methods rely on hazardous reagents like tri-n-octyltin azide [8,16], safer alternatives such as the use of sodium azide and zinc triflate have emerged [17].

In June 2018, the FDA was informed of a carcinogenic and genotoxic impurity, *N*-nitrosodimethylamine (NMDA), in valsartan. Nitrosamines can form via nitrosating reactions between amines (2°, 3°, or 4°) and nitrous acid. This prompted widespread recalls of sartan-based drugs which were found to have unacceptable levels of nitrosamine impurities. Regulatory agencies, such as the FDA and EMA, thus called for the development of safer, greener synthetic routes [18].

Green chemistry, or sustainable chemistry, aims to design chemical processes and products that reduce environmental impacts while maximizing resource efficiency. The 12 principles proposed by Anastas and Warner [19] include renewable feedstocks, waste prevention, energy efficiency, and a reduction in hazardous or toxic substances. These principles are especially relevant to pharmaceutical synthesis, where traditional methods are often dependent on toxic reagents and energy-intensive processes and generate significant amounts of waste. By incorporating green chemistry principles, the environmental footprint of processes can be reduced and their efficiency and economic viability can be improved [20].

Natural catalysts derived from renewable resources, such as seaweed extracts, offer sustainable alternatives and address environmental and economic challenges in pharmaceutical manufacturing. Although ligand-free Pd catalysts and ionic liquids used in Suzuki–Miyaura reactions have demonstrated potential as greener alternatives, scalability and waste reduction remain challenges [20]. The use of seaweed extracts exemplifies the concept of renewable feedstocks. Seaweed-derived materials are abundant, biodegradable, and rich in functional biomolecules that serve as polyphenols (reducing agents), polysaccharides (stabilizers), and catalysts [21]. These extracts may replace synthetic, toxic reagents, and the reaction conditions can be optimized to enhance atom economy and recyclability. For example, Pd nanoparticles synthesized from seaweed extracts have been used successfully in high-yielding, scalable, and recyclable cross-coupling reactions [22,23,24].

Given the limitations of traditional methods, this study aimed to develop a cost-effective, more environmentally friendly approach for synthesizing key losartan intermediates (Scheme 1). By employing biologically derived Pd nanoparticles synthesized from aqueous *Sargassum incisifolium* extracts, this study achieved the gram-scale synthesis of a biphenyl moiety while avoiding the use of problematic solvents such as DMF which could lead to nitrosamine formation.

## 2. Results

A practical, more environmentally friendly approach to the overall synthesis of losartan (**9**, Scheme 1), commencing with the key biaryl coupling of 2-bromobenzonitrile (**1**) and 4-methylphenylboronic acid (**2**) catalyzed by sustainably synthesized PdNPs, was developed. Following the bromination of **3** to form 2-(4-bromomethylphenyl)benzonitrile (**4**), the coupling of the biaryl intermediate (**4**) with imidazole carbaldehyde (**6**), followed by tetrazole formation, led to the synthesis of losartan (**9**) as the final product.

### 2.1. The Synthesis and Characterization of the PdNP Nanocatalyst

**Figure 1:**
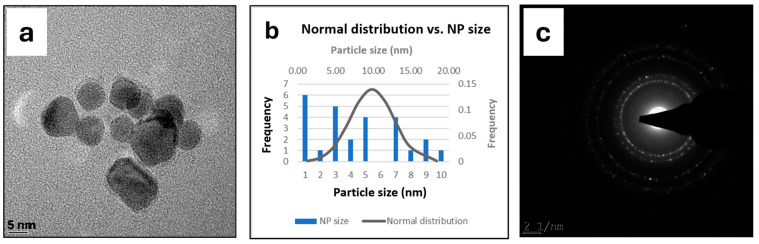
TEM (**a**), size distribution (**b**), and SAED (**c**) analyses of the PdNPs.

The aqueous *S. incisifolium* extract used to synthesize the PdNPs was prepared and analyzed by UV/visible, IR, and NMR spectroscopy and found to contain polysaccharides and polyphenols as its major and minor constituents, respectively, as previously reported [25,26]. In addition, antioxidant and free radical scavenging assays were carried out (Figure S1), and the data were consistent with the findings obtained by Mmola et al. (2016) and Mubaiwa et al. (2023) [25,26]. The total phenolic content and total reducing power of the aqueous extracts were found to be 79 μg/mg GAEs (Gallic Acid Equivalents) and 96 μg/mg AAEs (Ascorbic Acid Equivalents) per mg of dried seaweed, respectively, while the radical scavenging power was found to be ~60% at 10 mg/mL (Figure S1). The PdNP catalyst was prepared by heating the aqueous seaweed extract with a 0.1 M solution of K_2_PdCl_4_ at 80 °C for 24 h. After the reaction, the PdNPs were characterized by UV/visible spectroscopy, ICP-OES, and TEM. Due to the limited quantity of material, powder XRD analysis could not be performed; instead, the catalyst was employed as a solution following washing and centrifugation. TEM analysis revealed that the nanoparticles were spherical and polycrystalline (Figure 1a), with an average size of between 8 and 10 nm, as shown by the normal distribution curve (Figure 1b). The crystallinity of the PdNPs was further confirmed by the SAED patterns, which displayed distinct diffraction rings indicative of their polycrystalline nature (Figure 1c).

The formation of the PdNPs using aqueous *S. incisifolium* extracts was facilitated by the rich polyphenol and polysaccharide content present in the seaweed, as previously reported by Mmola et al. (2016) and Mubaiwa et al. (2023) [25,26]. Polyphenols act as natural reducing agents, converting Pd(II) ions to Pd(0), while polysaccharides, particularly sulphated fucoidans, serve as stabilizing and capping agents, preventing nanoparticle aggregation. The presence of the polyphenols and polysaccharides ensured the formation of uniformly sized, stable PdNPs, as confirmed through TEM and SAED analyses. These biomolecules therefore played an important role in the reduction and stabilization processes necessary to enable the green synthesis of catalytically active nanoparticles under mild conditions.

### 2.2. Synthesis of Biphenylcarbonitrile (**3**)

**Scheme 2:**
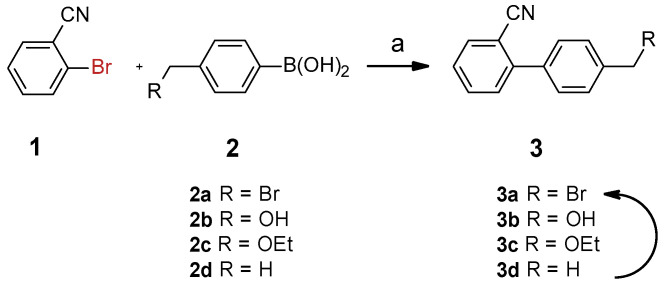
Suzuki–Miyaura reaction of 2-bromobenzonitrile and various boronic acids (**2a**–**d**). Conditions: solvent (20 mL), aryl halide (1 mmol), phenylboronic acid (1.5 mmol), base (2 mmol), catalyst (1 mol%).

Using the synthesized nanocatalyst, 4′-(bromomethyl)-biphenylcarbonitrile (**3a**, Scheme 2) was prepared by coupling 2-bromobenzonitrile (**1**) with three potential boronic acids (**2a**–**d**) and evaluated. The most direct route to **3a** involved the boronic acid **2a**. While the reaction with **2a** initially appeared successful, HPLC (Figure S3) and NMR (Figure S4) analysis revealed the formation of side products, including analogues of benzyl alcohol **3b** and ethyl ether **3c**, due to benzylic bromide substitution. Switching the solvent from aqueous ethanol to aqueous acetone improved the selectivity for the benzyl alcohol **3b** but did not yield **3a**. Attempts to synthesize **3a** in DMF were successful; however, the goal was to prioritize the use of environmentally friendly solvent systems like acetone/H_2_O. Furthermore, **3a** was found to be unstable, prompting the use of an alternative boronic acid, **2d**, which produced the *p*-tolyl benzonitrile **3d** efficiently. The use of boronic acids **2b** and **2c** also provided good yields of their respective biaryl products, and these intermediates could be converted to **3a** using PBr_3_ [27], NBS/AIBN [17], or NBS/light [28,29].

The reaction needed to produce **3d** was successfully scaled to 5.5 mmol (~1 g), achieving a 98% yield. ICP-OES analysis confirmed the absence of residual palladium in the final biaryl product.

#### 2.2.1. Reaction Kinetics

The kinetics of the Suzuki–Miyaura reaction needed to produce the biaryl intermediate **3d** were analyzed using 0.3 mol% and 1 mol% PdNPs as catalysts. The conversion of starting material **1** was monitored by HPLC, and the data revealed that the reaction rate and efficiency increased with catalyst loading (Table 1). At 0.3 mol% PdNPs, the reaction achieved a 94% conversion after 24 h, with a turnover number (TON) of 37,170, indicating good catalytic efficiency (Figure S6a). In contrast, using 1 mol% PdNPs resulting in a faster reaction, reaching 91% conversion within 3 h and a TON of 8897 (Figure S6b). The conversion of the starting material was also monitored by NMR at 25 °C using 1 mol% Pd. The reaction achieved a full conversion after 102 h with a TON of 10,877. Liu et al. (2012) produced a ligand-free catalyst with a TON efficiency of ~9000 and conversion in 5 h at a comparable loading of 0.3 mol% [30]. In this case, the recyclability was limited to two cycles, with the conversion dropping to 70% due to catalyst agglomeration. Although the approach Liu et al. (2012) established also aligned with green chemistry principles, the method employed in this work integrates a renewable feedstock (seaweed extract), and it avoids catalyst agglomeration [30]. Siamaki et al. (2011) employed a palladium–graphene catalyst at 0.5 mol% to achieve a 95% conversion in 6 h with a TON of ~20,000 [31]. This catalyst maintained a 92% conversion over three cycles but started to show moderate deactivation due to aggregation. The green PdNP catalyst employed therefore demonstrated good catalytic performance in terms of its TON, efficiency at lower loadings, and recyclability (Section 2.2.2) compared to other examples from the literature.

The corresponding HPLC data (Figure S6) illustrate a rapid decline in the concentration of 2-bromobenzonitrile (**1**), accompanied by the formation of **3d** as the main product, alongside two minor side products (with retention times of 10.9 and 2.7 min). The reaction kinetics for the conversion of benzonitrile **1** to the biaryl product **3d** at PdNP loadings of 0.3 mol% and 1 mol% are shown in Figure S7. At a 0.3 mol% Pd loading (Figure S7a), the reaction followed first-order kinetics, with a rate constant of 9.06 × 10^−4^ min^−1^. However, second-order kinetics with two distinct rates were observed for the 1 mol% Pd catalyst loading (Figure S7b). The initial, steep, increase in the kinetics over the first 3 h revealed a rate increase of 1.29 × 10^−2^ min^−1^, followed by a slower second rate of 2.40 × 10^−3^ min^−1^. The results therefore demonstrate the influence of catalyst loading on the reaction order and rate. The reaction progressed more slowly at a lower catalyst loading, while at 1 mol%, there was an initial rapid reaction followed by a marked decrease in the rate. This may have been due to substrate depletion or catalyst deactivation. However, the recyclability studies (Section 2.2.2) indicated that the former was more likely to have been the case.

The kinetics of the biaryl coupling reaction using 1 mol% Pd were also studied using ^1^H NMR in the same mixed solvent system of deuterated acetone and D_2_O (1:1) at 25 °C. This differed from the conditions of the HPLC study, which was conducted at 35 °C. The NMR spectra (Figure S8) show the disappearance of the *p*-tolylboronic acid (**2d**) and the simultaneous formation of product **3d** over 102 h. The signals (H_1_, H_2_, and H_3_) corresponding to the boronic acid moiety of product **3d** were integrated and plotted (Figure S10) to monitor the reaction progress. The excess unreacted **2d** is denoted by *. Additionally, the signals for the benzonitrile (**1**) starting material overlapped significantly with other peaks, rendering them unsuitable for kinetic evaluation. The plot (Figure S9) demonstrated zero-order behaviour with two distinct reaction rates. The initial rate (Rate_1_, t = 0 to t = 24 h) was faster, while the later rate (Rate_2_, t = 24 to t = 102 h) decreased significantly. This decline likely reflects a reduction in the availability of the reactants, as supported by the recyclability study results, although partial catalyst deactivation cannot be ruled out. The high R^2^ values indicate a good fit, which is consistent with the reaction mechanism being independent of the reactant concentration in these time intervals.

The reaction rates observed in the NMR study were slower than those in the HPLC study. This difference was likely due to the lower reaction temperature (25 °C vs. 35 °C), which reduced the reaction rate according to the Arrhenius equation. Additionally, the solubility of the reactants may have been reduced at lower temperatures, while the use of deuterated solvents may also have altered the catalyst activity, further affecting the reaction kinetics. The data also demonstrate the applicability of NMR for in situ kinetic studies, providing insights into reaction progression and product formation under mild, environmentally friendly conditions.

#### 2.2.2. Catalyst Recyclability

**Figure 2:**
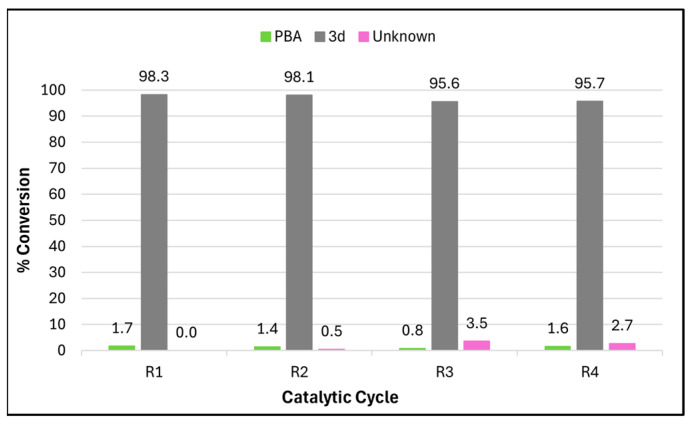
Percentage conversion data obtained from the HPLC chromatograms used in the recyclability study for 4 cycles using the PdNP catalyst (at 1 mol%). Data are given for the starting material (phenylboronic acid (PBA, used in excess)), **3d**, and an unknown product.

The recyclability of the PdNP catalyst was assessed through recovery via centrifugation and washing it with acetone, followed by the addition of fresh starting materials. This study (Figure 2) demonstrated its robustness and efficiency over four catalytic cycles (R1–R4). As shown in Figure S10, the HPLC chromatograms revealed the high product purity of **3d**, starting at 97.6% in the first cycle and declining slightly to 96.9% by the fourth cycle. Figure 2 shows the consistent conversion rates for the desired product **3d**, ranging from 98.3% to 95.7%, with a minimal increase in the amount of unreacted boronic acid (**2d**, PBA) from 1.7% to 3.5% and a slight increase in an unknown side product from 2.0 to 2.7%.

The results suggest that while the catalyst’s activity decreased marginally due to gradual deactivation or loss during recovery, it remained effective across multiple cycles.

### 2.3. Synthesis of 2-(4-Bromomethylphenyl)benzonitrile (**4**)

The Wohl–Ziegler reaction is a selective benzylic bromination of alkylarenes using *N*-bromosuccinimide (NBS), which avoids the use of hazardous reagents like chlorine and bromine [8]. Traditionally, toxic solvents such as carbon tetrachloride or dichloromethane and radical initiators like AIBN [8,17] have been employed despite their carcinogenicity, ozone-depleting properties, and environmental hazards [28]. In contrast, this study achieved the efficient bromination of **3d**, producing brominated *p*-tolylbenzonitrile (**4**) with a 72% yield under green conditions, using a 9W LED lamp as a sustainable energy source and acetonitrile as a safer alternative solvent at 70 °C, in just 2 h (Figure S11). Madasu et al. (2012) achieved an 80% yield of brominated product **4**, though the use of 1,3-dibromo-5,5-dimethylhydantoin (DBDMH)/AIBN in dichloromethane at reflux for 24 h was required [8]. This approach significantly reduced the reaction time and eliminated the need for toxic solvents and reagents.

### 2.4. Synthesis of Losartan Intermediates **6**–**8**

Processes for the *N*-alkylation of the commercially available 2-butyl-4-chloro-5-(hydroxymethyl)imidazole **5** and **4** include biphasic systems with sodium or potassium hydroxide as their base and long reaction times. We initially explored using a method proposed by Remuzzi et al. for this reaction (2003) [32]; however, a relatively low yield (~39%) was obtained using dry ethanol and NaOMe at 40 °C over 4 h for the required product **8**. This reaction was attempted under various reaction conditions as shown in Tables S2 and S3, where the use of NaOMe in DMF at 40 °C provided a yield of 52% in 4 h. However, the oxidation of the primary alcohol and 2-butyl-4-chloro-1H-imidazole-5-carbaldehyde (**6**, Figure S12) following a method described by Miller and Hoerrner (2003) [33] increased the acidity of the N-H, improving the yield of **7** significantly (~70%). In comparison, Madasu et al. (2012) [8] reported a 71% yield using dimethylacetamide and potassium carbonate as their base. HCl gas and ammonia under high pressure have also previously been employed to produce the carbaldehyde intermediate; however, the environmental implications have resulted in their restricted application in industry [17]. Starting with valeronitrile and methanol in the presence of acetyl chloride at 0–5 °C and atmospheric pressure, Shuangxia et al. (2015) were able to produce the carbaldehyde **6** (Figure S12) in two steps with an 82% yield [17]. In our work, the crude carbaldehyde product **7** (Figure S13) was then reduced smoothly to the alcohol **8** (Figure S14) using sodium borohydride and methanol, resulting in a 90% yield. However, the reaction proceeded with about a 73% yield over two steps from the production of the imidazole **5** to the alcohol product **8** (Figures S14 and S15) using ethanol as the solvent.

### 2.5. Synthesis of Tetrazole (Losartan, **9**)

The final step in the synthesis was the conversion of the benzonitrile **8** to a tetrazole ring. Tetrazoles have commonly been prepared via the cycloaddition of azides and nitriles [16]. To avoid using hazardous organotin reagents, as have been employed in previous reactions [8,17], several catalysts, including zinc bromide [12], zinc triflate [17], ammonium chloride, and proline, were investigated. In this process, the amount of sodium azide was limited in an attempt to avoid the generation of highly toxic and explosive hydrazoic acid with an acidic workup [8]. The final product, **9**, was obtained using a method described by Demko and Sharpless (2001) [12] with a 77% yield. Vigorous stirring and the addition of 2-propanol an hour after starting the reaction appeared to be essential. The product was revealed to have identical chromatographic behaviour (Figure S16) and NMR spectra (Figures S17 and S18) to those of the reference standard. In addition, no nitrosamine impurities were detected via HPLC. Thus, this process can avoid creating environmental pollution through the use of organic solvents with reduced reaction times and lower temperature requirements.

## 3. Discussion

In summary, this study presented a practical and more environmentally friendly approach to the production of losartan (Scheme 1), beginning with the high-yielding (98%) Suzuki–Miyaura biaryl coupling of 2-bromobenzonitrile (**1**) and 4-methylphenylboronic acid (**2d**), catalyzed by PdNPs derived from a renewable seaweed-based source. A comparison of common biaryl coupling strategies in the literature on losartan synthesis is provided in Table S4 (Supporting Information), which highlights the environmental advantages of our method, especially in terms of the high catalyst recyclability, the mild reaction conditions, and the avoidance of toxic reagents relative to traditional approaches such as Stille, phosphine-ligated Suzuki, and Ullmann-type coupling.

Following the bromination of **3d** using an NBS/LED light source to form **4**, the coupling of this biaryl intermediate with imidazole carbaldehyde (**6**) was followed by tetrazole formation, and losartan (**9**) was obtained as the final product with a 27% overall yield. While several synthetic steps in the synthetic route to losartan synthesis included several well-established procedures reported in the literature and patents, the primary focus was the development of a more sustainable approach to the biaryl coupling step. Although the use of alternatives such as proline, ammonium chloride, and zinc triflates was briefly evaluated in the downstream steps, this resulted in significantly lower yields and was not pursued further.

Our approach had several advantages, including the use of benign solvents, mild reaction conditions, and a catalyst system that is recyclable, efficient at low loadings, and consistent with green chemistry principles. The methodology eliminated the need for the use of organotin reagents, HCl gas, AIBN, and other hazardous substances reported in the patent literature on high-yielding reactions for losartan production, offering a cleaner, safer alternative. Thus, the hazardous reagents and solvents typically used in traditional synthesis were replaced with more environmentally benign alternatives, and the PdNPs showed favourable catalytic performance (in terms of their TON, recyclability, and efficiency at lower catalyst loadings). While the process was effective and reproducible at the gram scale, further studies are needed to assess its scalability, particularly in terms of PdNP recovery, the consistency of the nanoparticle quality, and the stability of sensitive intermediates under scaled-up conditions. Nonetheless, this strategy provides a promising foundation for the future development of scalable, eco-friendly pharmaceutical manufacturing.

## 4. Materials and Methods

### 4.1. General Experimental Details

All chemicals and solvents were purchased from Merck and used without purification. All reactions were monitored by TLC, and NMR spectra were acquired using a 400 MHz Avance IIIHD Nanobay spectrometer (Bruker, Rheinstetten, Germany) equipped with a 5 mm BBI probe at 298 K using standard 1D and 2D NMR pulse sequences in DMSO-*d*_6_ or CDCl_3_. All chemical shifts (δ) are given in ppm with reference to the residual solvent signal, while the coupling constants (*J*) are in Hz. An Agilent (Santa Clara, CA, USA) 1200 Series HPLC system equipped with a quaternary pump, photodiode array detector (PDA), in-line degasser, and PC with Chemstation OpenLab software was used for the determination of purity and kinetics and recyclability studies. HRESIMS (high-resolution electrospray ionization) was performed at the Central Analytical Facility at Stellenbosch University using the MassLynx software (version 4.1) on a Waters Synapt G2 spectrometer (Milford, MA, USA). The ionization source used was an ESI^+^, with a cone voltage of 15 V. An ICap 6200 Inductively Coupled Plasma-Atomic Emission Spectrometer (ICP-AES, Thermo, Waltham, MA, USA) was used to determine the Pd content. The instrument was calibrated and validated using NIST (National Institute of Standards and Technology, Gaithersburg, MD, USA) traceable standards purchased from Inorganic Ventures (Christiansburg, VA, USA). Sample morphology and elemental analyses were performed using Transmission Electron Microscopy (TEM) and energy-dispersive X-ray spectroscopy (EDX). TEM images were collected using a Tecnai G2 20 field emission gun (FEG, FEI, Hillsboro, OR, USA) TEM, operated in the bright field mode at an accelerating voltage of 200 kV. EDX spectra were collected using a liquid nitrogen-cooled lithium-doped silicon detector (EDAX, Hillsboro, OR, USA). The size of the nanoparticles in the TEM images was determined using ImageJ software (https://imagej.net/ij/, accessed on 26 July 2024).

### 4.2. Synthetic Procedures

All the steps in the key biaryl coupling procedure were based on modified procedures from the literature. The use of alternative reagents such as proline, ammonium chloride, and Zn(OTf)_2_ was explored in the tetrazole formation step but led to significantly reduced yields under the reaction conditions employed. These were not included in the final synthesis route.

#### 4.2.1. Collection of *Sargassum incisifolium* and Preparation and Characterization of Aqueous Extract

The brown seaweed *Sargassum incisifolium* was collected in 2019 from the Western Cape coast of South Africa. An aqueous extract (AE) was prepared through extraction from the seaweed (10 g wet weight) using milli-Q water at 100 °C for 1 h. The aqueous extract was freeze-dried and stored at −20 °C until further use. The total phenolic content, reducing power, and radical scavenging power of the aqueous seaweed extract sample were determined using the methods described by Topiwala et al. (2014) [34]. In addition, the ^1^H and HSQC NMR data for the sample were also recorded as per the procedure described by Mmola et al. (2016) [25].

#### 4.2.2. Synthesis of Palladium Nanoparticles

The nanoparticles (PdNPs) were prepared as follows: 750 μL of a 0.1 M solution of a palladium salt (K_2_PdCl_4_) was added to 25 mL of a 1 mg/mL solution of the aqueous *S. incisifolium* extract to obtain a final 1:19 metal/extract ratio. The solution was then refluxed at 80 °C for 24 h. After completion, the aqueous *S. incisifolium* extract-capped PdNPs were analyzed by TEM and UV/visible spectroscopy. The concentration of palladium in the NP samples was determined to be 340.458 nm using ICP-AES. Briefly, 600 μL of the PdNP sample reaction solution was made up to 50 mL (~6 ppm) and analyzed. The Pd concentration for the PdNP sample was determined to 5.38 ppm.

#### 4.2.3. The General Procedure for the Suzuki–Miyaura Coupling Reactions (Scheme 2: **3a**–**3d**)

2-bromobenzonitrile aryl halide (**1**, 1.0 mmol), 4-methylphenylboronic acid (**2d**, 1.5 mmol), K_2_CO_3_ (2.0 mmol), PdNPs (1 mol% (0.986 mg Pd, 9.27 μmol Pd) or 0.3 mol% (0.269 mg Pd, 2.53 μmol Pd)), and acetone/H_2_O in a 1:1 ratio (20 mL) were introduced into a 50 mL round-bottomed, two-necked flask equipped with a stirrer bar and a silicon stopper. The reaction mixture was stirred at 35 °C. Compounds **3b** and **3c** were prepared similarly using the respective boronic acids. Alternatively, 4-bromomethylphenylboronic acid (**2a**) could be used to prepare **3b** and **3c** by employing ethanol/H_2_O as a solvent system, while **3b** could be obtained using acetone/H_2_O as a solvent system. Compound **4** was produced similarly, using 4-bromomethylphenylboronic acid (**2a**) in DMF only. For HPLC analysis, 100 μL aliquots were drawn at various time intervals, 1.9 mL of acetonitrile was added, and the samples were centrifuged at 2000 rpm. Subsequently, 1.5 mL of the supernatant was removed and added to an HPLC vial for analyses. The final reaction products (at 24 h) were then extracted using dichloromethane and the organic layer was dried over anhydrous magnesium sulphate and concentrated under a low pressure. The target 2-(*p*-tolyl)benzonitrile product (**3d**) was purified by silica gel column chromatography using EtOAc/hexane (2:8) as the eluant to yield 188.2 mg (98.0%) for **3d**.

*Compound* **3a**: ^1^H NMR (400 MHz, CDCl_3_) δ 7.76 (dd, *J* = 7.7, 1.4 Hz, 1H), 7.64 (td, *J* = 7.7, 1.4 Hz, 2H), 7.57–7.48 (m, 7H), 7.45 (td, *J* = 7.6, 1.3 Hz, 2H), 4.54 (s, 3H); ^13^C NMR (101 MHz, CDCl_3_) δ 144.6, 138.2, 138.1, 133.7, 132.9, 129.9, 129.4, 129.1, 127.8, 118.5, 111.1, 32.8; MS *m*/*z* (TOF MS): 192.08 [(M-HBr)^+^]. Yield: 97.1%.
*Compound* **3b**: ^1^H NMR (400 MHz, CDCl_3_) δ 7.72 (dd, *J* = 7.7, 1.4 Hz, 1H), 7.61 (td, *J* = 7.7, 1.4 Hz, 1H), 7.54–7.37 (m, 7H), 4.67 (s, 2H); ^13^C NMR (101 MHz, CDCl_3_) δ 145.1, 141.5, 137.0, 133.6, 132.8, 129.86, 128.7, 127.4, 127.0, 118.7, 110.8, 64.2, 50.2, 15.0; MS *m*/*z* (TOF MS): 192.08 [(M-HOH)^+^]. Yield: 96.0%.
*Compound* **3c**: ^1^H NMR (400 MHz, CDCl_3_) δ 7.74 (dd, *J* = 7.8, 1.4 Hz, 1H), 7.62 (td, *J* = 7.7, 1.4 Hz, 1H), 7.57–7.37 (m, 6H), 4.56 (s, 2H), 3.58 (q, *J* = 7.0 Hz, 2H), 1.27 (t, *J* = 7.0 Hz, 3H); ^13^C NMR (101 MHz, CDCl_3_) δ 145.3, 139.3, 137.3, 133.8, 132.8, 130.1, 128.8, 128.1, 127.6, 118.8, 111.2, 72.3, 66.0, 15.3; MS *m*/*z* (TOF MS): 192.08 [(M-HOCH_2_CH_3_)^+^]. Yield: 98.2%.
*Compound* **3d**: ^1^H NMR (400 MHz, CDCl_3_) δ 7.74 (dd, *J* = 7.9, 1.4 Hz, 1H), 7.62 (td, *J* = 7.7, 1.4 Hz, 1H), 7.52–7.38 (m, 4H), 7.33–7.27 (m, 2H), 2.42 (s, 3H). ^13^C NMR (101 MHz, CDCl_3_) δ 145.4, 138.6, 135.2, 133.6, 132.7, 129.9, 129.4, 128.5, 127.2, 118.8, 111.1, 21.2; MS *m*/*z* (TOF MS): 192.08 [(M-H)^+^]. Yield: 98.0%.

#### 4.2.4. Recyclability Studies

The coupling reaction of aryl bromide (**1**) with 4-methylphenylboronic acid (**2d**) was carried out as described above, following the general procedure for Suzuki–Miyaura cross-coupling reactions. After 24 h, the reaction mixture was centrifuged, the supernatant removed, and the catalyst washed with H_2_O (1 × 10 mL), followed by acetone (1 × 10 mL), and centrifuged (at 2000 rpm) each time to collect the catalyst. A total of 1 mL of water was then added to the recovered catalyst, and this used in a further coupling experiment. The supernatant (100 μL) was added to an HPLC vial with 1.9 mL of acetonitrile and analyzed via HPLC. The remaining supernatant was extracted using DCM, dried over magnesium sulphate, and concentrated under a low pressure. The ^1^H NMR spectra were subsequently recorded in CDCl_3_ for each cycle.

#### 4.2.5. Synthesis of 2-[4-(Bromomethyl)phenyl]benzonitrile (**4**)

A common household lamp was used to provide the visible light energy necessary to homolytically cleave the Br-Br bond and initiate the radical cascade, as described by Marcos et al., 2020 [28]. The use of carbon tetrachloride and dichloromethane was avoided due to environmental issues, and thus acetonitrile was employed as the most suitable solvent with which to perform the reaction. The 2-(*p*-tolyl)benzonitrile (**3d**, 264 mg, 1.37 mmol) produced by the Suzuki–Miyaura coupling reaction was dissolved in 4 mL of acetonitrile in a 25 mL round bottom flask fitted with a condenser and stirrer bar. *N*-bromosuccinimide (243 mg, 1.37 mmol) and a further 4 mL of acetonitrile were added. The reaction mixture was heated to 70 °C, and the mixture was irradiated with a 9W LED (765 lumens, colour temperature of 4500 K) source for 2 h until the reaction was complete (as monitored by TLC). The acetonitrile was evaporated and the product washed with water. The dried reaction product was purified by silica gel column chromatography using EtOAc/hexane (2:8) as the eluant to yield 246.1 mg (72.0%) of **4**. The product was characterized using 1D and 2D NMR.

^1^H NMR (400 MHz, CDCl_3_) δ 7.76 (dd, *J* = 7.6, 1.4 Hz, 1H), 7.65 (td, *J* = 7.7, 1.4 Hz, 1H), 7.58–7.47 (m, 5H), 7.45 (td, *J* = 7.6, 1.3 Hz, 1H), 4.54 (s, 2H); ^13^C NMR (101 MHz, CDCl_3_) δ 144.6, 138.2, 138.1, 133.7, 132.9, 129.9, 129.4, 129.1, 127.8, 118.5, 111.1, 32.8; MS *m*/*z* (TOF MS): 192.08 [(M-HBr)^+^].

#### 4.2.6. Synthesis of 2-Butyl-4-chloro-1H-imidazole-5-carbaldehyde (**6**)

The method described by Miller and Hoerrner (2003) was followed with a few modifications [33]. 2-Butyl-4-chloro-5-(hydroxymethyl)imidazole (**5**) (750.1 mg) was weighed into a 100 mL round bottom flask equipped with a magnetic stirring bar. Toluene (10 mL) was added and the mixture stirred, followed by the addition of a sodium bicarbonate solution (1.00 g, 11.94 mmol in 10 mL of deionized water). The slurry was charged with solid iodine at 20 °C (2.03 g, 7.96 mmol), followed by solid TEMPO (62 mg, 0.398 mmol). The mixture was allowed to stir overnight (16 h) at 20 °C and then cooled to 5 °C. The mixture was diluted with EtOAc (10 mL) and quenched with aqueous sodium sulfite (Na_2_SO_3_, 501 mg in 5 mL deionized water). Additional EtOAc (10 mL) and deionized water (10 mL) were added and the mixture separated. The organic layer was washed with saturated aqueous KHCO_3_ (10 mL), followed by brine (10 mL). The washed organic layer was diluted to 50 mL and dried over Na_2_SO_4_ and then concentrated to 10 mL under pressure. The solution was allowed to crystallize, and the crystals were washed with cold toluene to yield 518.8 mg (69.9%) of **6**.

^1^H NMR (400 MHz, CDCl_3_) δ 9.61 (s, 1H), 3.09–2.54 (m, 2H), 1.81–1.69 (m, 2H), 1.44–1.31 (m, 2H), 0.92 (t, *J* = 7.3 Hz, 3H).

#### 4.2.7. Synthesis of 2-[4-[(2-Butyl-4-chloro-5-formyl-imidazol-1-yl)methyl]phenyl]benzonitrile (**7**)

The method described by Shuangxia et al. (2015) was followed with a few modifications [17]. Briefly, 2-butyl-4-chloro-1H-imidazole-5-carbaldehyde (**6**) (185.6 mg, 1 mmol) and K_2_CO_3_ (231.1 mg, 1.67 mmol) were added to a solution of 2-[4-(bromomethyl)phenyl]benzonitrile (**4**) (301.1 mg, 1.1 mmol) in DMF (5 mL) at ambient temperature. The resulting mixture was stirred and heated to 40 °C for 6 h. Water was added to the mixture and extraction was performed using a 1:1 EtOAc/hexane mixture (15 mL × 3); the combined organic phase was washed with water and saturated brine and concentrated under a low pressure. The product (**7**) was obtained as a viscous yellow liquid and used without purification, yielding 297.2 mg (79.0%). These reactions were also successful when using EtOH and isopropanol with various reaction times, and the results are presented in Table S3.

^1^H NMR (400 MHz, CDCl_3_) δ 9.75 (s, 1H), 7.74 (dd, *J* = 7.7, 1.4 Hz, 1H), 7.61 (dd, *J* = 7.7, 1.4 Hz, 1H), 7.52–7.39 (m, 4H), 7.20–7.12 (m, 2H), 5.60 (s, 2H), 2.71–2.57 (m, 2H), 1.76–1.63 (m, 2H), 1.41–1.24 (m, 2H), 1.23 (t, *J* = 7.1 Hz, 1H), 0.88 (t, *J* = 7.4 Hz, 3H).

#### 4.2.8. Synthesis of 2-N-butyl-4-chloro-l-[2′-cyanobiphenyl-4-yl) methyl]-5-(hydroxymethyl)-imidazole (**8**)

Method 1: A solution of 2-butyl-4-chloro-5-(hydroxymethyl)imidazole, **5** (0.5 mmol, 95 mg), in 1.5 mL of dry EtOH was added to NaOCH_3_ (0.9 mmol, 49.9 mg) in 2 mL EtOH. The resulting mixture was stirred at room temperature for 15 min, then 4′-bromomethyl-2-biphenylcarbonitrile (**4**) (0.6 mmol, 162.7 mg), dissolved in 1.5 mL of EtOH, was added to the reaction mixture, and it was subsequently stirred at 40 °C for 4 h. The solvent was removed under a low pressure and the remaining mixture diluted with water, followed by extraction using EtOAc (x 3). The organic layers were combined, washed with saturated brine, dried over NaSO_4_, filtered, and then concentrated. The crude product was further purified by silica gel column chromatography using mixtures of hexane/EtOAc to obtain the desired losartan intermediate **8** with a yield of 73.9 mg (39.0%). Various reaction conditions were tested and are given in Table S2.

Method 2: A method described by Shuangxia et al. (2015) was followed with a few modifications [17]. MeOH (2.4 mL) and toluene (1 mL) were added to a 25 mL vial containing the crude imidazole carbaldehyde **7** (435 mg) under a nitrogen atmosphere at ambient temperature. The reaction mixture was gently stirred and cooled to 0–5 °C; then, sodium borohydride (37.3 mg, 0.986 mmol) was added and the mixture was stirred for 10 min. The reaction was allowed to warm up to ambient temperature while it was stirred for 1 h. Then, the mixture was cooled to 10 °C, water (10 mL) was added to quench the excess sodium borohydride, and the reaction mixture was extracted using EtOAc (15 mL × 3). The combined organic phase was washed with water (20 mL) and brine (20 mL) and concentrated under a low pressure. The crude product **8** was purified by silica gel column chromatography using a mixture of EtOAc/hexane, yielding 339.6 mg (89.6%).

^1^H NMR (400 MHz, CDCl_3_) δ 7.75 (dd, *J* = 7.7, 1.4 Hz, 1H), 7.63 (td, *J* = 7.7, 1.4 Hz, 1H), 7.55–7.48 (m, 2H), 7.50–7.40 (m, 2H), 7.14–7.08 (m, 2H), 5.29 (s, 2H), 4.50 (s, 2H), 2.61–2.53 (m, 2H), 2.47 (s, 1H), 1.71–1.59 (m, 1H), 1.33 (h, *J* = 7.4 Hz, 2H), 0.86 (t, *J* = 7.3 Hz, 3H); MS *m*/*z* (TOF MS): 380.15 [(M + H)^+^].

#### 4.2.9. Synthesis of Losartan (**9**)

A slightly modified version of a procedure proposed by Demko and Sharpless (2001) was followed [12]. A mixture of compound **8** (349.8 mg, 0.921 mmol), ZnBr_2_ (228.3 mg, 1.014 mmol), NaN_3_ (67 mg, 1.031 mmol), and water (5 mL) was stirred vigorously at reflux. After 1 h, 2-propanol (2 mL) was added to the solution following some precipitation, and the reaction mixture remained at reflux for 24 h. Thereafter, HCl (3 N, 5 mL) and EtOAc (5 mL) were added to the reaction flask until no precipitate was observed. The organic layer was isolated and the aqueous layer extracted using 2 × 20 mL of EtOAc. The combined organic layers were evaporated, 40 mL of NaOH (0.25 N) added, and the mixture stirred for 30 min until all the precipitates dissolved and a suspension of zinc hydroxide formed. The suspension was filtered, and the solid phase was washed with 20 mL of 1 N NaOH. A total of 10 mL of 3 N HCl was added to the filtrate, and the mixture was stirred. The tetrazole precipitate was subsequently filtered and washed with 2 × 5 mL 3 N HCl and dried in an oven to yield the final product, **9**. (Yield: 300.9 mg, 77.3%.)

^1^H NMR (400 MHz, MeOD) δ 7.55–7.45 (m, 2H), 7.44–7.38 (m, 2H), 7.11 (d, *J* = 8.3 Hz, 2H), 6.91 (d, *J* = 8.3 Hz, 2H), 5.25 (s, 2H), 4.42 (s, 2H), 2.57 (t, *J* = 7.9 Hz, 2H), 1.58–1.48 (m, 2H), 1.36–1.24 (m, 2H), 0.86 (t, *J* = 7.3 Hz, 3H); MS *m*/*z* (TOF MS): 424.14 [(M + 2H)^+^].

## Figures and Tables

**Table 1:** Conversion and turnover (TON) data for the Suzuki–Miyaura reaction converting 2-bromobenzonitrile (**1**) to the biaryl intermediate **3d** using PdNP catalysts at 0.3 mol% and 1 mol% loadings.

Catalyst	Time/h	Conversion, % ^a^	TON ^b^
PdNPs (0.3 mol%)	24	94	37,170
PdNPs (1 mol%)	3	91	8897
PdNPs (1 mol%) *	102 *	100 *	10,877 *

Reactions were performed using 1 mmol of 2-bromobenzonitrile (**1**), 1.5 mmol of 4-methyl phenylboronic acid (**2d**), 2 mmol of K_2_CO_3_, and 20 mL of solvent (acetone/H_2_O: 1:1) at 35 °C and under air. ^a^ Reaction conversion in % was calculated through HPLC based on relative quantities of starting material, desired product, and two minor side products. * Reaction conversion in % was calculated through NMR based on relative quantity of **1** and desired product **3d** in deuterated acetone and D_2_O at 25 °C. ^b^ TON was calculated using following equation: TON = mole of converted 2-bromobenzonitrile (**1**)/mole of Pd catalyst × 100%.

## Data Availability

The original contributions presented in this study are included in the article/Supplementary Materials. Further inquiries can be directed to the corresponding author(s).

## Supplementary Materials

The following supporting information can be downloaded at https://www.mdpi.com/article/10.3390/molecules30112314/s1: Table S1. Gradient profile for the biaryl intermediate and losartan/NMDA samples. Figure S1. Results obtained for total polyphenolic content (GAE μg/mL), total reducing power (AAE μg/mL), and radical (DPPH) scavenging power (%) measured at 760, 700, and 520 nm for aqueous *Sargassum incisifolium* seaweed extract. Figure S2. Calibration curve obtained from the HPLC data (λ = 242 nm) for 2-bromobenzonitrile (**1**) over the range of 3 μM to 4 mM. The LOQ was determined to be below 3 μM. Figure S3. HPLC chromatograms (λ = 242 nm) obtained using method 1 for the target compound 4-bromomethyl-biphenylcarbonitrile (**3a**) and the additional biaryl products **3b**–**d**. Figure S4. ^1^H NMR (400 MHz, CDCl_3_) spectra of the target compound 4-bromomethyl-biphenylcarbonitrile (**3a**) and the additional biaryl products **3b**–**d**. Figure S5. HR TOFMS spectra for compounds **3a**–**d** in the positive ion mode. Figure S6. HPLC percentage height data (λ = 242 nm) obtained for the conversion of 2-bromobenzonitrile (**1**) to product **3d** using (a) 0.3 mol% and (b) 1 mol% of the PdNP catalyst at 35 °C. The retention times for **1**, **3d**, **unknown 1,** and **unknown 2** were 3.4, 5.6, 10.9 and 2.7 min, respectively. Figure S7. HPLC kinetics data (λ = 242 nm) obtained for the conversion of 2-bromobenzonitrile (**1**) to product **3d** using (a) 0.3 mol% (first-order kinetics) and (b) 1 mol% (second-order kinetics) of the PdNP catalyst at 35 °C. Two rates were obtained (rate_1 for t = 0 to t = 3 h and rate_2 for t = 3 to 1440 h) for the 1 mol% PdNP catalyst. The trendline equation and R^2^ values are indicated for each. Figure S8. ^1^H NMR kinetics spectra at various time intervals (t = 0 to t = 102 h) for **3d** in D_2_O/acetone-*d*_6_ at 298 K. * indicates the starting material, *p*-tolylboronic acid (**2d**), which was used in excess (1.5 eq.). Figure S9. Zero-order kinetics obtained from the ^1^H NMR (400 MHz, acetone/*d*_6_:D_2_O (1:1), 298 K) data for H_5_, H_9_, and H_12_ for the formation of compound **3d**. There were two rates obtained for each of the protons (rate_1 (t = 0 to 24 h) and rate_2 (t = 24 to 102 h)). The trendline equation and R^2^ values are indicated for each. Figure S10. HPLC chromatograms (obtained using method 1) used for the recyclability study for **3d** employing the PdNP catalyst: (a) 1st cycle, (b) 2nd cycle, (c) 3rd cycle, and (d) 4th cycle. The percentage purity of the compound is given below the respective retention times. Figure S11. ^1^H NMR (400 MHz, CDCl_3_) data for compound **4**. Table S2. Percentage yields for the coupling of imidazole, **5,** and biaryl, **4**. Figure S12. ^1^H NMR (400 MHz, CDCl_3_) data for compound **6**. Table S3. Optimization of coupling of imidazole carbaldehyde, **6,** and biaryl, **4**. Figure S13. ^1^H NMR (400 MHz, CDCl_3_) data for compound **7**. Figure S14. ^1^H NMR (400 MHz, CDCl_3_) data for compound **8**. Figure S15. HR TOF-MS data for compound **8** obtained in the positive ion mode. Figure S16. HPLC chromatograms (obtained using method 2) for the synthesized (a) and reference standard (b) losartan (**9**) at λ = 250 nm. Figure S17. ^1^H NMR (400 MHz, MeOD) data for compound **9** (losartan). Figure S18. HR TOF-MS data for compound **9** obtained in the positive ion mode. Table S4. Comparison of biaryl coupling strategies in Losartan (**9**) synthesis. Refs. [13,32,35,36] are cited in the Supplementary Materials.
