# Supplementary material for: Balancing Yields and Sustainability: An Eco-Friendly Approach to Losartan Synthesis Using Green Palladium Nanoparticles
Source: Molecules. 2025 May 25;30(11):2314. doi: 10.3390/molecules30112314 (PMC12155655; doi:10.3390/molecules30112314)
Supplement: Supplementary file 1 [file molecules-30-02314-s001.zip › molecules-3585849-supplementary.pdf]

Supplementary information

Balancing Yield and Sustainability: An Eco-Friendly Approach to Losartan Synthesis Using Green Palladium Nanoparticles

Edith M. Antunes[1], Yusuf A. Adegoke[2], Sinazo Mgwigwi[2], John J. Bolton[3], Sarel F. Malan[2], Denzil R. Beukes[2]*

[1]Chemistry Department, University of the Western Cape, Private Bag X17, Bellville, 7535, South Africa; E-Mail: ebeukes@uwc.ac.za (E.M.A)

[2]School of Pharmacy, University of the Western Cape, Private Bag X17, Bellville, 7535, South Africa; E-Mail: adegoke.adeyemi@gmail.com (Y.A.A), 3761240@myuwc.ac.za (S.M), sfmalan@uwc.ac.za (S.F.M), dbeukes@uwc.ac.za (D.R.B)

[3]Department of Biological Sciences, University of Cape Town, Private Bag X3, Rondebosch, 7701, South Africa; E-Mail: john.bolton@uct.ac.za (J.J.B)

*Author to whom correspondence should be addressed; E-Mail: dbeukes@uwc.ac.za (D.R.B); Tel.: +27-21-959-2352; Fax: +27-21-959-3407.

Table of Contents:

1. Experimental details 3

1.1. HPLC method 3

Table S1. Gradient profile for the biaryl intermediate and Losartan/NMDA samples 4

2.1 Results 4

2.1 Characterisation of the aqueous seaweed extract 4

Figure S1. Results obtained for total polyphenolic content (GAE μg/mL), total reducing power (AAE μg/mL) and radical (DPPH) scavenging power (%) assays measured at 760, 700 and 520 nm for the *Sargassum incisifolium* seaweed aqueous extract. 4

2.2 Synthesis and characterisation of Losartan (9) and its intermediates 5

Figure S2. Calibration curve obtained from HPLC data (λ = 242 nm) for 2-bromobenzonitrile (1) over the range of 3 μM to 4 mM. The LOQ was determined to be below 3 μM. 5

Figure S3. HPLC chromatograms (λ = 242 nm) using method 1 for the target compound 4-bromomethyl-biphenylcarbonitrile (**3a**) and the additional biaryl products **3b - d**. 5

Figure S4. ^{1}H NMR (400 MHz, $CDCl_3$) spectra of the target compound 4-bromomethyl-biphenylcarbonitrile (**3a**) and the additional biaryl products **3b - d**. 6

Figure S5. HR TOFMS spectra for compounds **3a - d** in the positive ion mode. 6

Figure S6. HPLC percentage height data (λ = 242 nm) obtained for the conversion of 2-bromobenzonitrile (**1**) to product **3d** using a) 0.3 mol% and b) 1 mol% of the PdNP catalyst at

35 °C. The retention times for **1**, **3d**, **unknown 1** and **unknown 2** are 3.4, 5.6, 10.9 and 2.7 min, respectively. 7

Figure S7. Kinetics HPLC data (λ = 242 nm) obtained for the conversion of 2-bromobenzonitrile (**1**) to product **3d** using a) 0.3 mol% (first order kinetics) and b) 1 mol% (second order kinetics) of the PdNP catalyst at 35 °C. Two rates were obtained (rate_1 for t = 0 to t = 3 h and rate_2 for t = 3 to 1440 h) for the 1 mol% PdNP catalyst. The trendline equation and R^2 values are indicated for each. 7

Figure S8. ^{1}H NMR kinetics spectra at various time intervals (t = 0 to t = 102 h) for **3d** in D_2O:Acetone-d_6 at 298K. *indicates the starting material *p*-tolylboronic acid (**2d**) which was used in excess (1.5 eq.). 8

Figure S9. Zero order kinetics obtained from the ^{1}H NMR (400 MHz, Acetone-d_6:D_2O (1:1), 298K) data for H_5, H_9 and H_{12} for the formation of compound **3d**. There are two rates obtained for each of the protons (rate_1 (t = 0 to 24 h) and rate_2 (t = 24 to 102 h)). The trendline equation and R^2 values are indicated for each. 8

Figure S10. HPLC chromatograms (using method 1) for the recyclability study: a) 1st cycle, b) 2nd cycle, c) 3rd cycle, and d) 4th cycle, for **3d** employing the PdNP catalyst. Percentage purity of the compound is given below the respective retention times. 9

Figure S11. ^{1}H NMR (400 MHz, $CDCl_3$) data for compound **4**. 9

Table S2. Percentage yields for the coupling of imidazole **5** and biaryl **4** 10

Figure S12. ^{1}H NMR (400 MHz, $CDCl_3$) data for compound **6**. 10

Table S3. Optimization of coupling of imidazole carbaldehyde **6** and biaryl **4** 11

Figure S13. ^{1}H NMR (400 MHz, $CDCl_3$) data for compound **7**. 11

Figure S14. ^{1}H NMR (400 MHz, $CDCl_3$) data for compound **8**. 12

Figure S15. HR TOF-MS data for compound **8** in the positive ion mode. 12

Figure S16. HPLC chromatograms (using method 2) for the synthesized (a) and reference standard (b) losartan (**9**) at λ = 250 nm. 13

Figure S17. ^{1}H NMR (400 MHz, MeOD) data for compound **9** (Losartan). 13

Figure S18. HR TOF-MS data for compound **9** in the positive ion mode. 14

Table S4. Comparison of biaryl coupling strategies in Losartan (**9**) synthesis 14

1. Experimental details

All chemicals and solvents were purchased from Merck and used without purification. NMR spectra were acquired on a 400 MHz Avance IIIHD Nanobay spectrometer (Bruker, Rheinstetten, Germany) equipped with a 5 mm BBO probe at 333 K using standard 1D and 2D NMR pulse sequences in MeOD, DMSO-d_6 or $CDCl_3$. All chemical shifts (δ) are given in ppm with reference to the residual solvent signal and coupling constants (J) are in Hz and chemical shift values are reported ppm. An Agilent 1200 Series HPLC system equipped with a quarternary pump, photodiode array detector (PDA), in-line degasser, and a desktop PC with the ChemStation OpenLab software was used for the determination of purity, kinetics and recyclability studies. HRESIMS (high-resolution electrospray ionisation) data were recorded at the Central Analytical Facility at Stellenbosch University using a Waters Synapt G2 spectrometer (Milford, MA, USA). The ionisation source was an ESI^+, with a cone voltage 15 V. An ICap 6200 Inductively Coupled Plasma-Atomic Emission Spectrometer (ICP-AES, Thermo, Waltham, MA, USA), used to determine the Pd content, was determined by the Central Analytical Facility at Stellenbosch University. The instrument was calibrated and validated using NIST (National Institute of Standards and Technology, Gaithersburg, MD, USA) traceable standards purchased from Inorganic Ventures (Christiansburg, VA, USA). Sample morphology and elemental analyses were accomplished using Transmission Electron Microscopy (TEM) and Energy Dispersive X-ray spectroscopy (EDX). TEM images were collected using a Tecnai G2 20 field-emission gun (FEG, FEI, Hillsboro, OR, USA) TEM, operated in bright field mode at an accelerating voltage of 200 kV. EDX spectra were collected using a liquid nitrogen cooled lithium doped silicon detector (EDAX, Hillsboro, OR, USA). The size of nanoparticles in the TEM images was determined using ImageJ software.

1.1. HPLC method

Separate methods were used to determine 1) the NMDA content / Losartan purity and 2) the kinetic/recyclability studies used in producing the biaryl intermediate (**3**). Milli-Q water and Acetonitrile were employed as the mobile phase with 0.1% formic acid. The solvents were filtered and degassed by membrane filtration through a 0.45 um HVLP Millipore filter (Bedford, MA, U.S.A) prior to use. The effect of decreasing the polarity of the solvent system was assessed by increasing the acetonitrile content by 10% at the various steps in the gradient programme. The flow rate was modified between 0.8 mL/min to 0.5 mL/min with the latter deemed to be the most suitable for both methods.

The final chromatographic parameters were as follows:

Column: Waters X-Terra, 5 µm, 4.6 x 150 mm
Column Temp: Room temperature

Mobile Phase A: H_2O (0.1% Formic acid)
Mobile Phase B: Acetonitrile (0.1% Formic acid)
Flow Rate: 0.5 mL/min
Detector: PDA at 250 nm, 220 nm, 230 nm, 242 nm, 268 nm
Injection Volume: 20 μL

Table S1. Gradient profile for the biaryl intermediate and Losartan/NMDA samples

**Method 1: Biaryl intermediate (3)**			**Method 2: Losartan (8)/NMDA**		
**Time (min)**	**% A**	**% B**	**Time (min)**	**% A**	**% B**
0.00	30	70	0.00	95	5
3.00	30	70	1.00	95	5
10.00	0	100	3.00	80	20
11.00	0	100	5.00	80	20
11.10	30	70	20.00	0	100
**15.00**	30	70	22.00	0	100
			22.10	95	5
			**25.00**	95	5

The retention times of the reference compounds at these chromatographic conditions were found to be 3.45 min and 5.67 min for the 2-bromobenzonitrile (**1**) and the main biaryl intermediate product (**3a** - **d**), respectively using method 1 (Figure S3); while the retention times for NMDA and Losartan (**9**) were at 5.6 min and 16.7 min (using method 2, Table S1), respectively.

2.1 Results

2.1 Characterisation of the aqueous seaweed extract

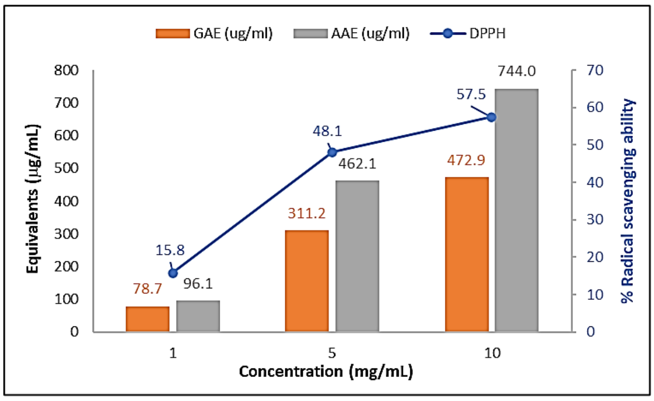

Figure S1. Results obtained for total polyphenolic content (GAE μg/mL), total reducing power (AAE μg/mL) and radical (DPPH) scavenging power (%) assays measured at 760, 700 and 520 nm for the *Sargassum incisifolium* seaweed aqueous extract.

2.2 Synthesis and characterisation of Losartan (9) and its intermediates

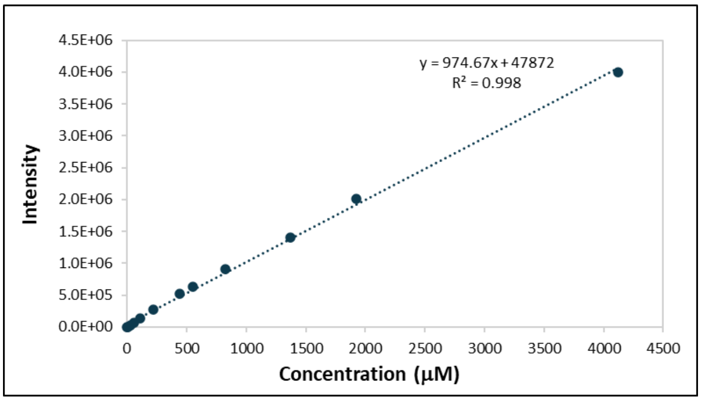

Figure S2. Calibration curve obtained from HPLC data (λ = 242 nm) for 2-bromobenzonitrile (1) over the range of 3 μM to 4 mM. The LOQ was determined to be below 3 μM.

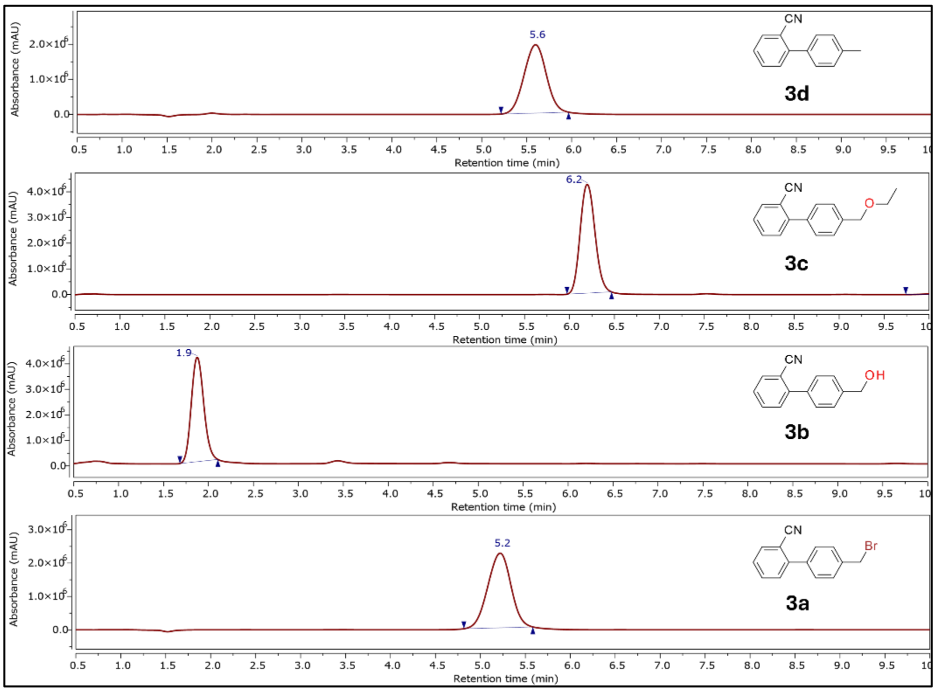

Figure S3. HPLC chromatograms (λ = 242 nm) using method 1 for the target compound 4-bromomethyl-biphenylcarbonitrile (**3a**) and the additional biaryl products **3b - d**.

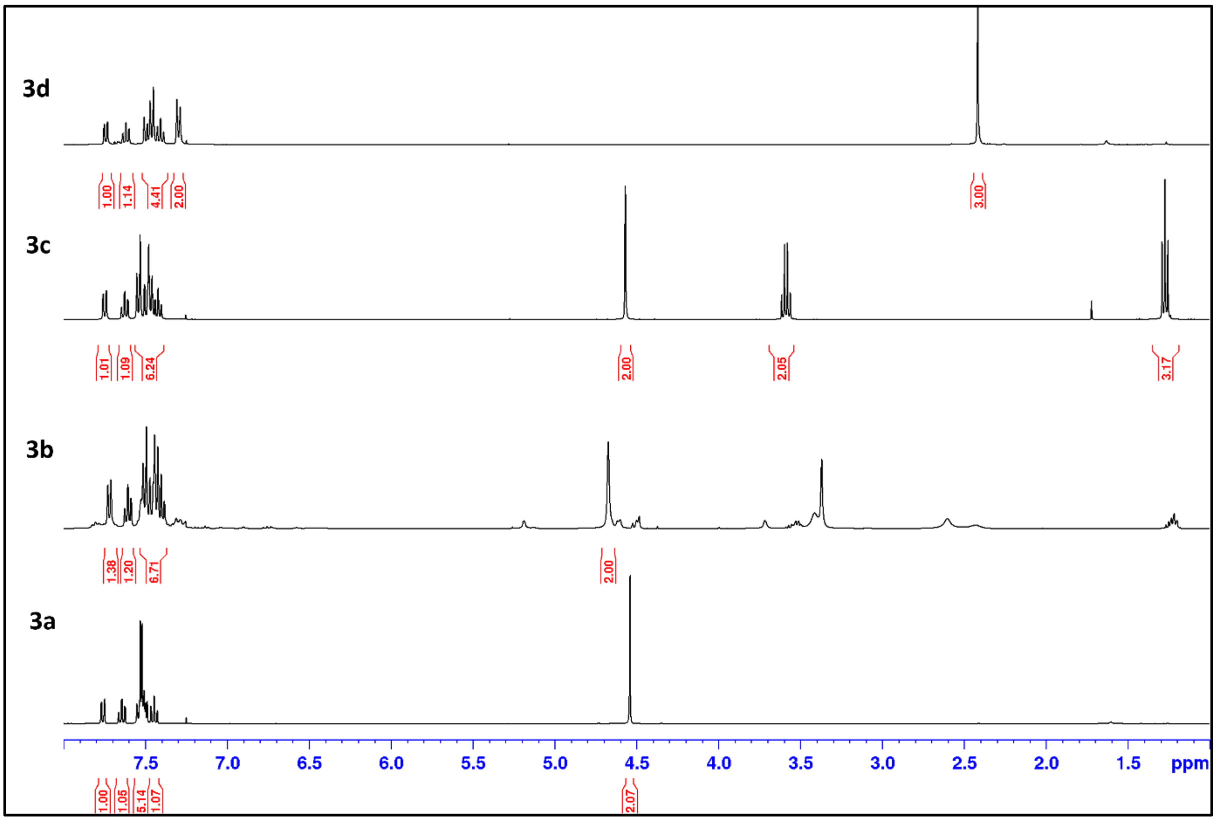

Figure S4. ^{1}H NMR (400 MHz, $CDCl_3$) spectra of the target compound 4-bromomethyl-biphenylcarbonitrile (**3a**) and the additional biaryl products **3b - d**.

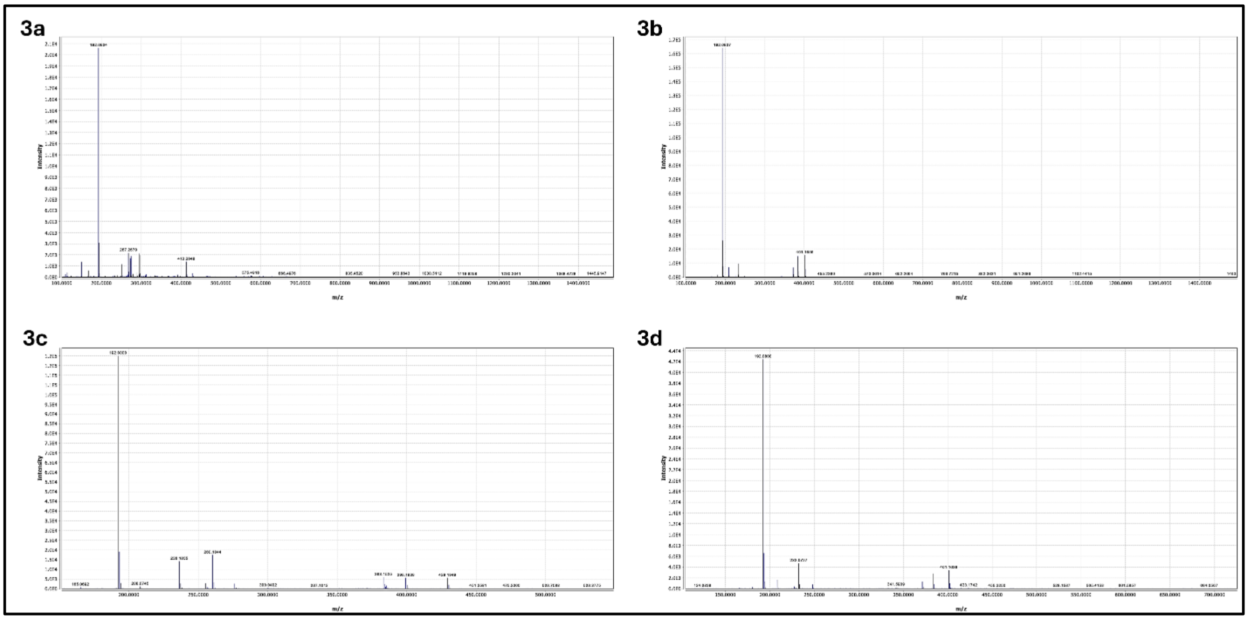

Figure S5. HR TOFMS spectra for compounds **3a - d** in the positive ion mode.

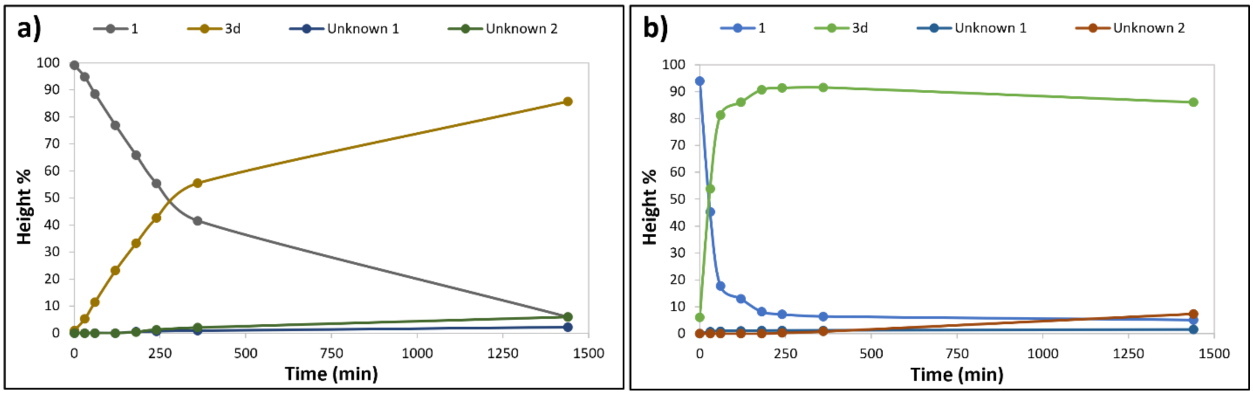

Figure S6. HPLC percentage height data (λ = 242 nm) obtained for the conversion of 2-bromobenzonitrile (**1**) to product **3d** using a) 0.3 mol% and b) 1 mol% of the PdNP catalyst at 35 °C. The retention times for **1**, **3d**, **unknown 1** and **unknown 2** are 3.4, 5.6, 10.9 and 2.7 min, respectively.

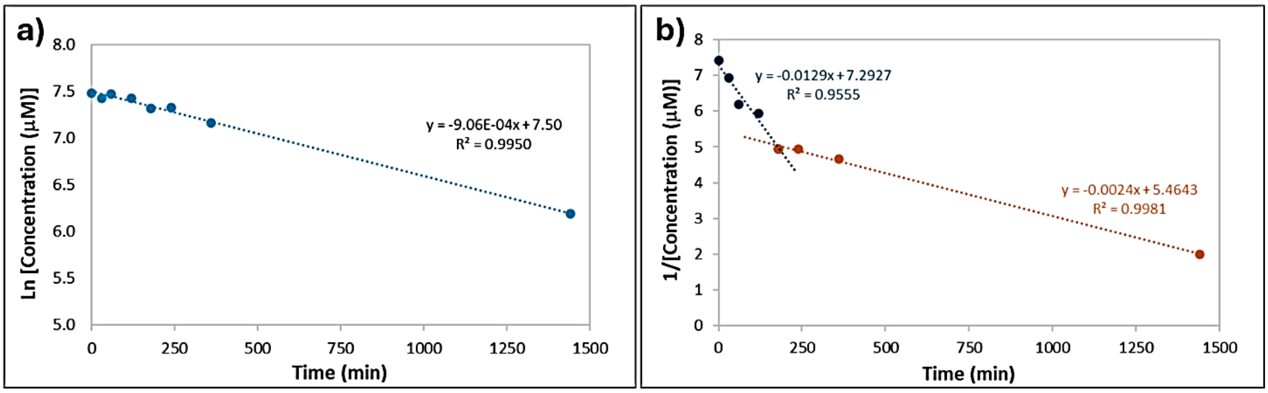

Figure S7. Kinetics HPLC data (λ = 242 nm) obtained for the conversion of 2-bromobenzonitrile (**1**) to product **3d** using a) 0.3 mol% (first order kinetics) and b) 1 mol% (second order kinetics) of the PdNP catalyst at 35 °C. Two rates were obtained (rate_1 for t = 0 to t = 3 h and rate_2 for t = 3 to 1440 h) for the 1 mol% PdNP catalyst. The trendline equation and R^2 values are indicated for each.

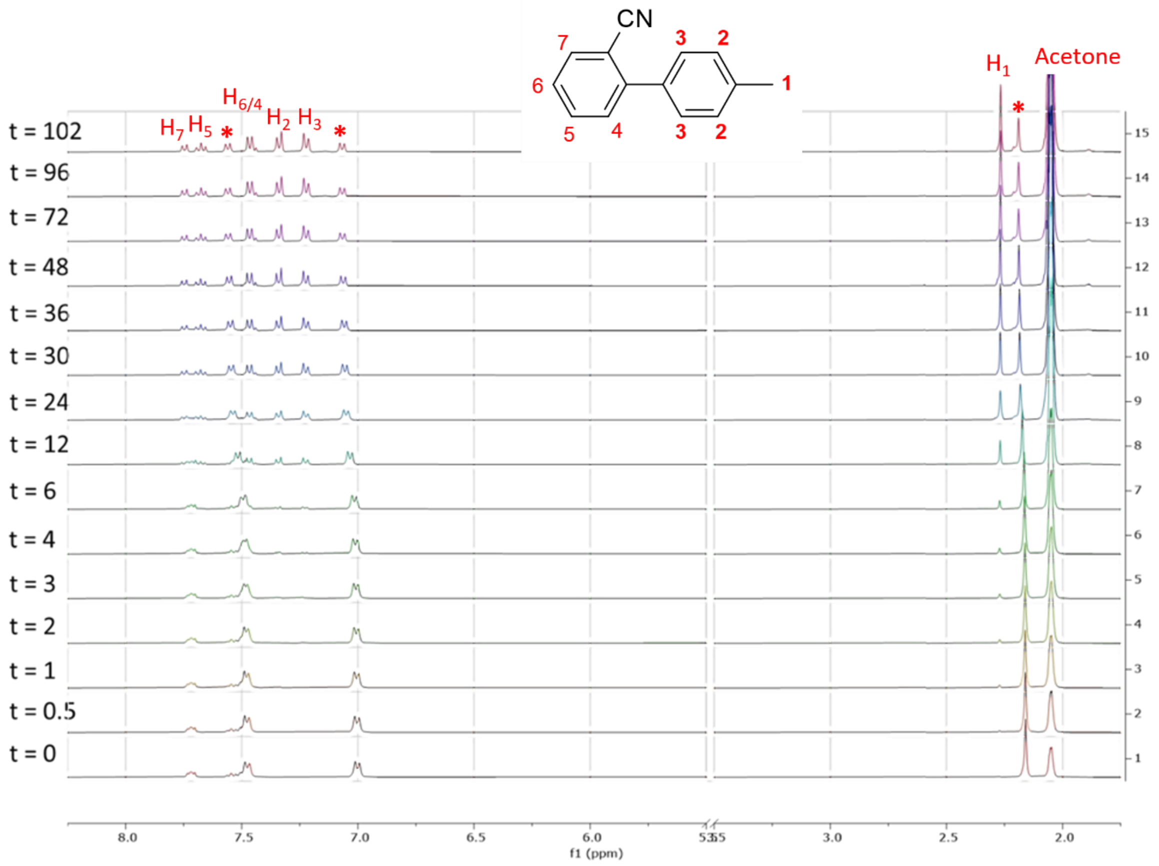

Figure S8. ^1H NMR kinetics spectra at various time intervals (t = 0 to t = 102 h) for **3d** in D_2O:Acetone-d_6 at 298K. *indicates the starting material *p*-tolylboronic acid (**2d**) which was used in excess (1.5 eq.).

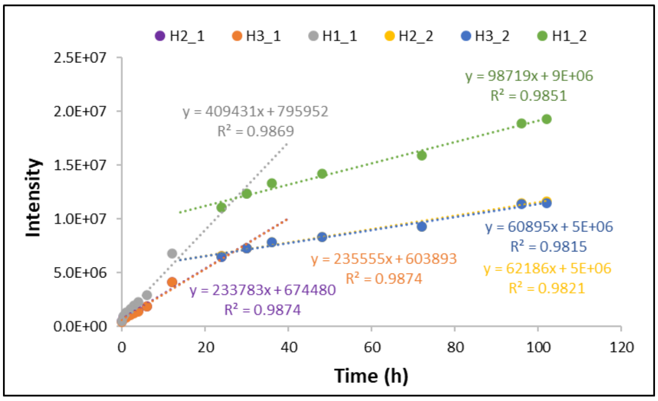

Figure S9. Zero order kinetics obtained from the ^1H NMR (400 MHz, Acetone-d_6:D_2O (1:1), 298K) data for H_5, H_9 and H_{12} for the formation of compound **3d**. There are two rates obtained for each of the protons (rate_1 (t = 0 to 24 h) and rate_2 (t = 24 to 102 h)). The trendline equation and R^2 values are indicated for each.

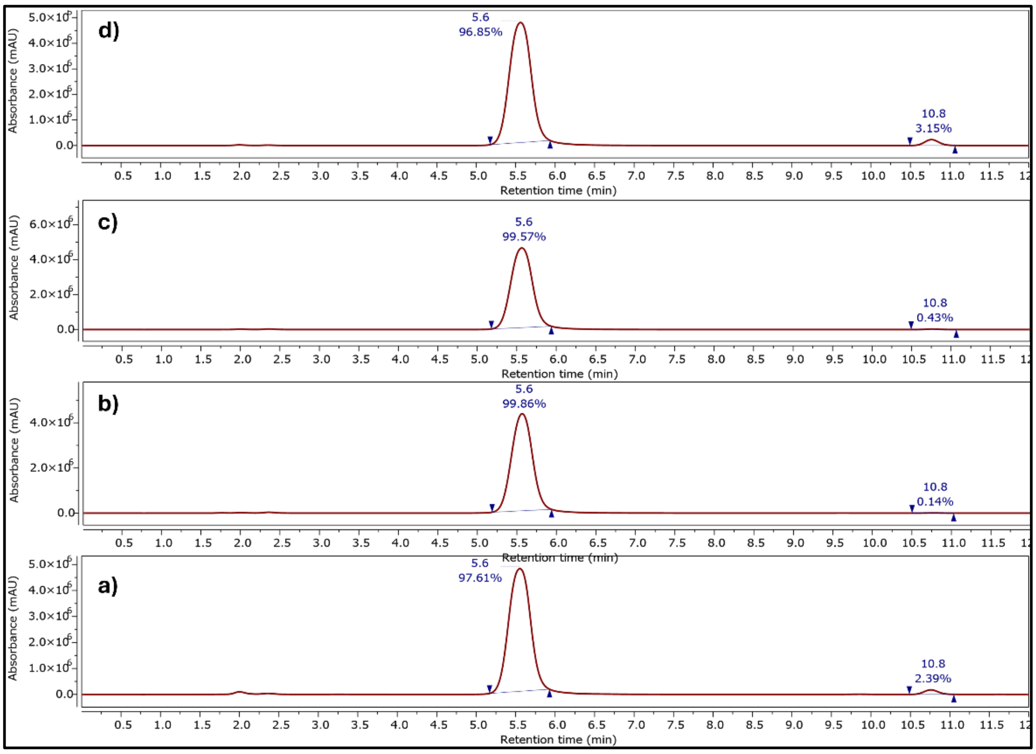

Figure S10. HPLC chromatograms (using method 1) for the recyclability study: a) 1st cycle, b) 2nd cycle, c) 3rd cycle, and d) 4th cycle, for **3d** employing the PdNP catalyst. Percentage purity of the compound is given below the respective retention times.

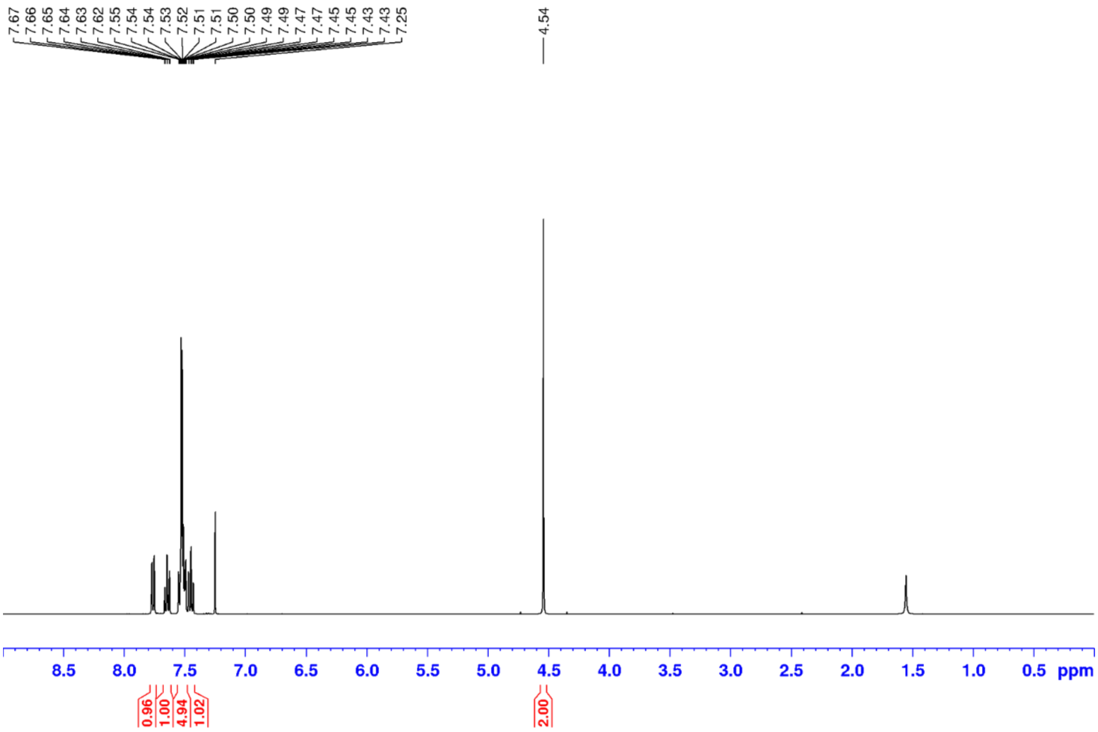

Figure S11. ^{1}H NMR (400 MHz, $CDCl_3$) data for compound **4**.

Table S2. Percentage yields for the coupling of imidazole **5** and biaryl **4**

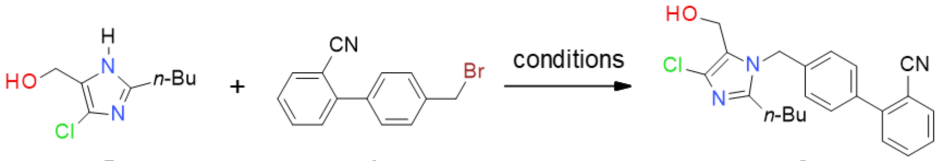

5 **4** **8**

Entry	Conditions	% Isolated yield for 8
Lit[1]	NaOMe, DMF, 40 °C, 4 h	52.0
A	NaOMe, dry EtOH, 40 °C, 4 h	39.0
B	K_2CO_3, DMF, 40 °C, 24 h	8.3

[1]Remuzzi *et al.* (2003)

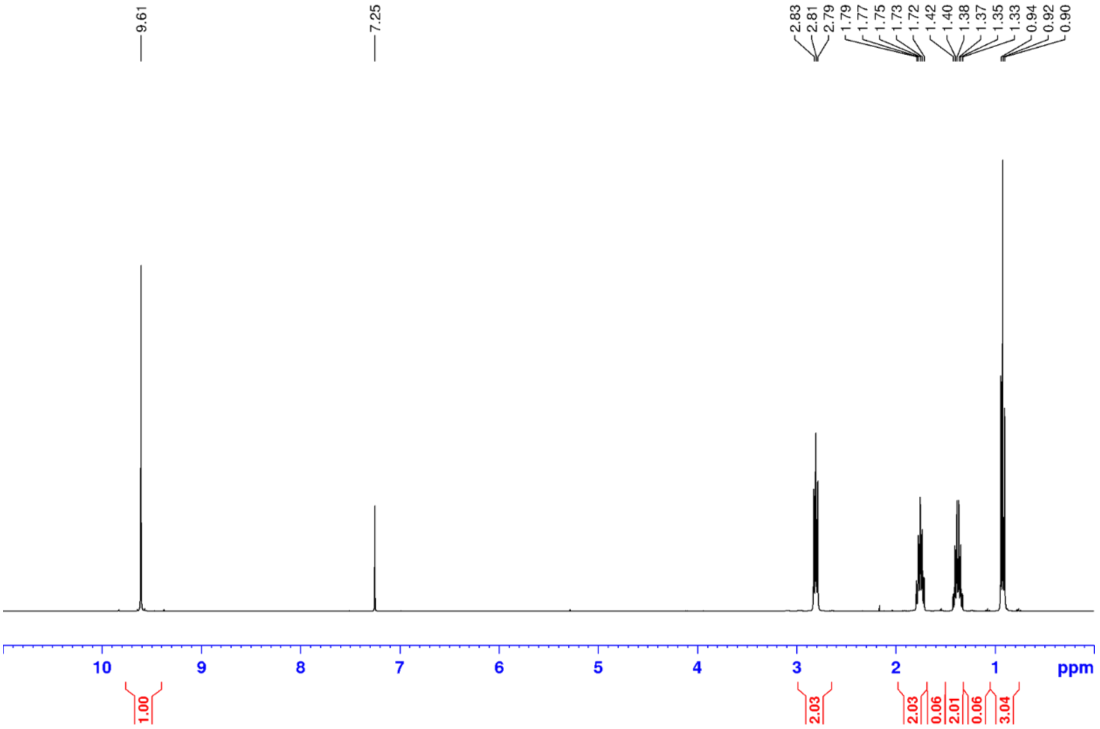

Figure S12. ^{1}H NMR (400 MHz, $CDCl_3$) data for compound **6**.

Table S3. Optimization of coupling of imidazole carbaldehyde **6** and biaryl **4**

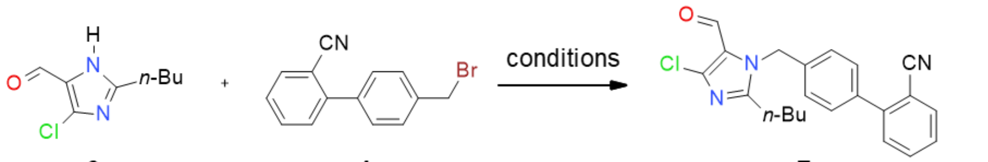

6 **4** **7**

Entry	Conditions	% Isolated yield for 7
Lit[1]	K_2CO_3, DMF, 40 °C, 6 h	79.0
A	K_2CO_3, EtOH, 40 - 45 °C, 6 h	67.0
B	K_2CO_3, EtOH, 40 - 45 °C, 24 h	72.9
C	K_2CO_3, EtOH, reflux, 6 h	73.2
D	K_2CO_3, EtOH, reflux, 24 h	49.5
E	K_2CO_3, 2-PrOH, reflux, 24 h	60.3

[1]Remuzzi *et al.* (2003)

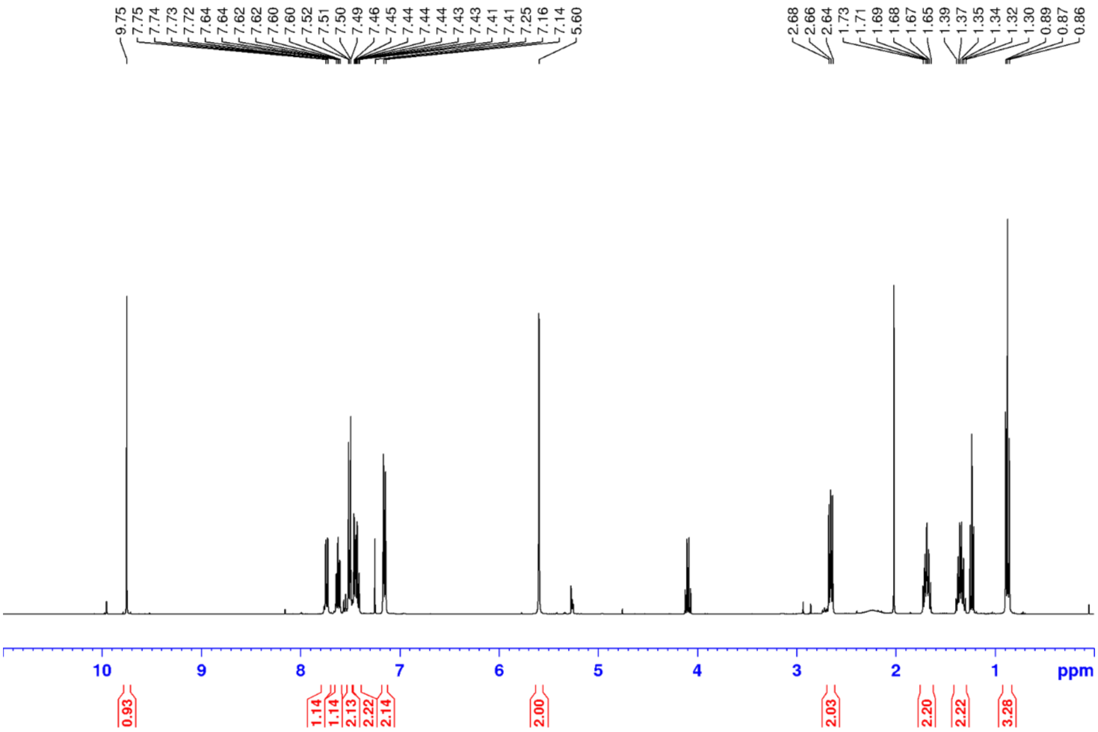

Figure S13. ^{1}H NMR (400 MHz, $CDCl_3$) data for compound **7**.

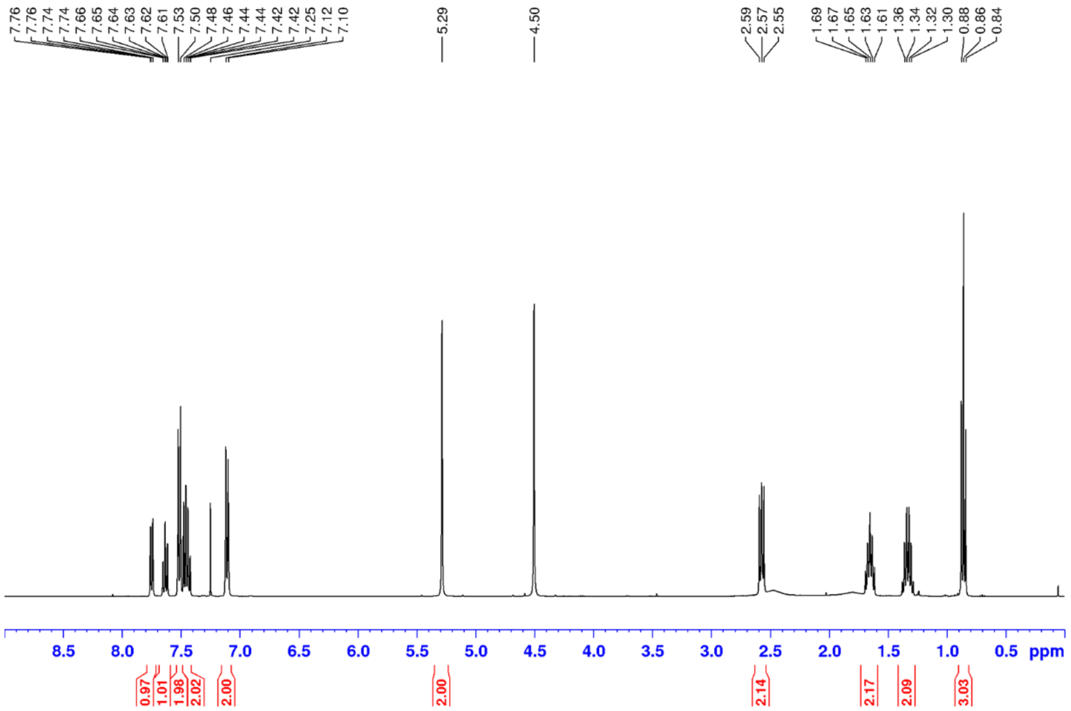

Figure S14. ^{1}H NMR (400 MHz, $CDCl_3$) data for compound **8**.

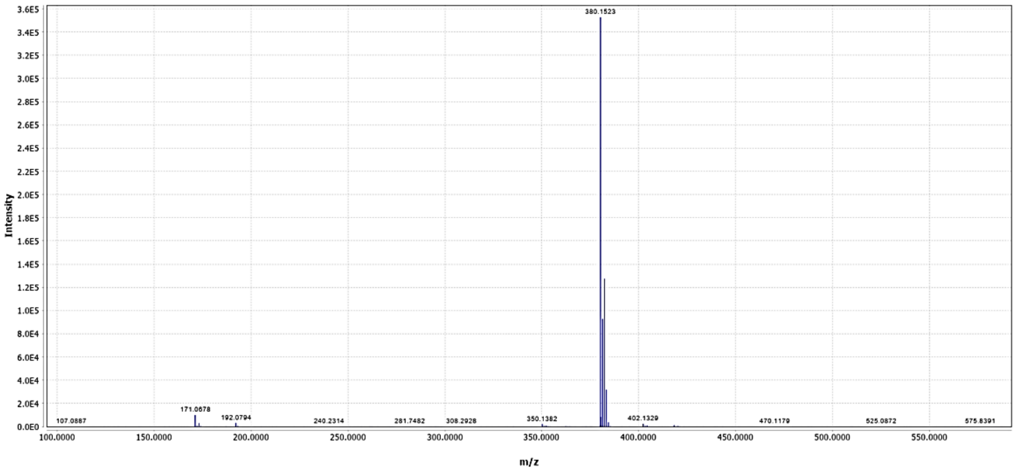

Figure S15. HR TOF-MS data for compound **8** in the positive ion mode.

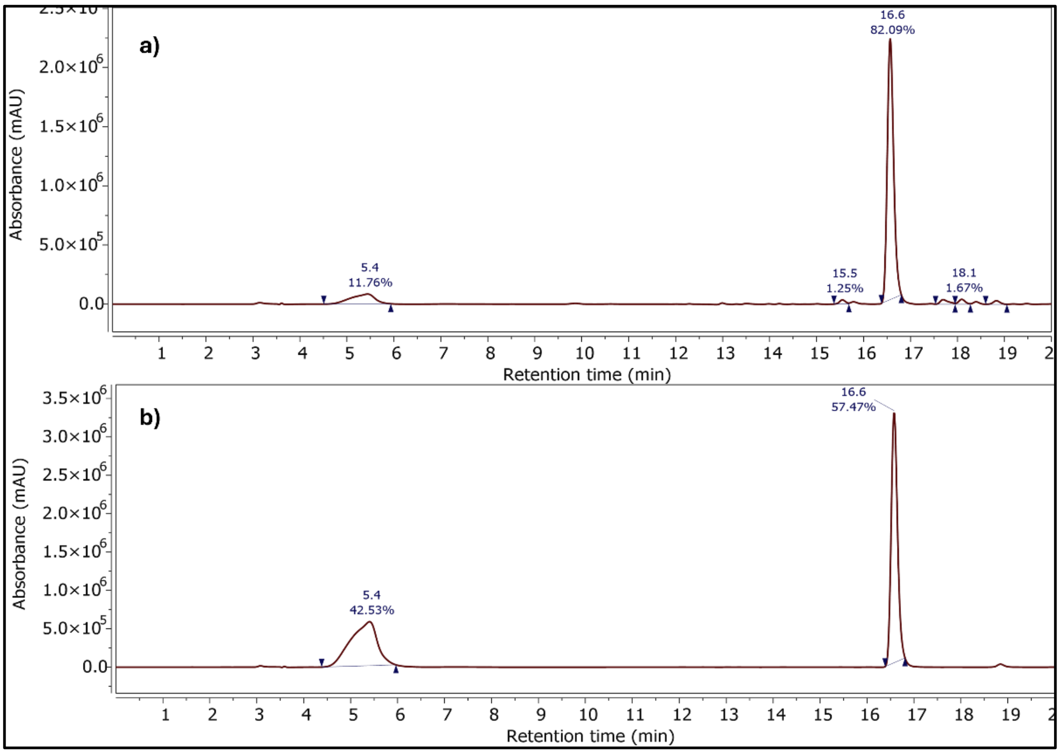

Figure S16. HPLC chromatograms (using method 2) for the synthesized (a) and reference standard (b) losartan (**9**) at λ = 250 nm.

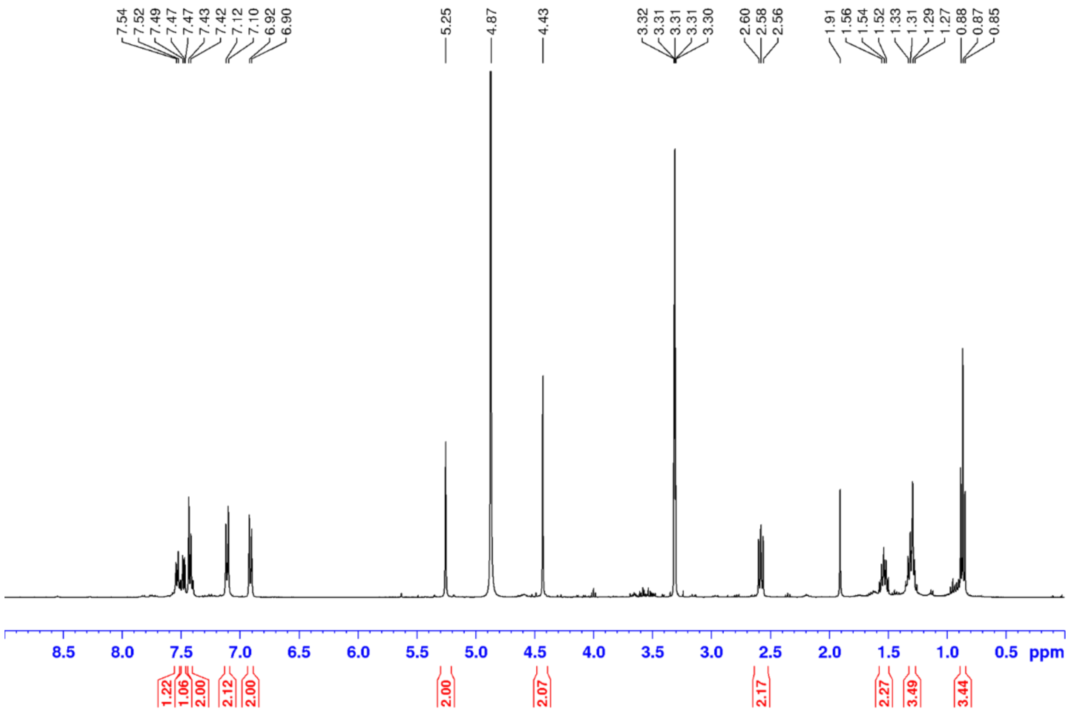

Figure S17. ^{1}H NMR (400 MHz, MeOD) data for compound **9** (Losartan).

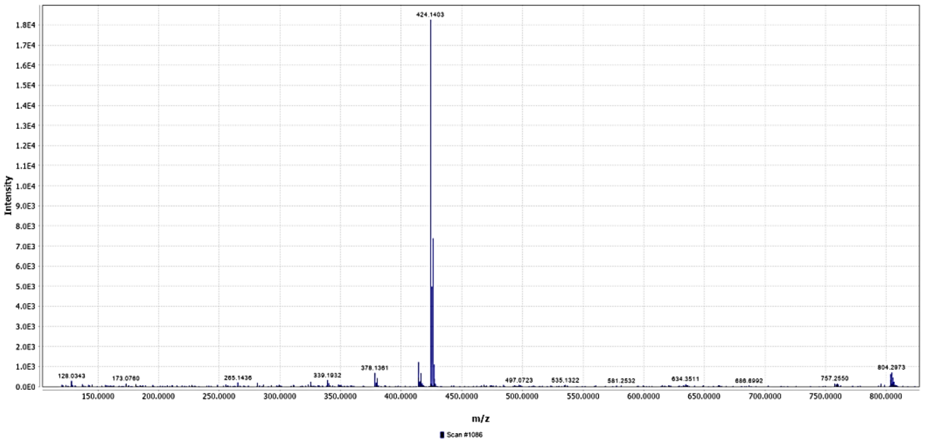

Figure S18. HR TOF-MS data for compound **9** in the positive ion mode.

Table S4. Comparison of biaryl coupling strategies in Losartan (**9**) synthesis

**Method**	**Catalyst Type**	**Typical Conditions**	**Environmental Concerns**	**Yield Range (Biaryl Step)**	**Sustainability Aspects**
Stille coupling[2]	Pd(0) with organotin reagent	Anhydrous, toxic solvents, heat	High toxicity, organotin waste, difficult removal	80–90%	Poor (non-green reagents, toxic waste)
Traditional Suzuki–Miyaura coupling[3]	$Pd(PPh_3)_4$ or $PdCl_2$(dppf)	Base, organic solvent (e.g., DMF, THF), phosphine ligands	Air-sensitive ligands, triphenylphosphine oxide waste	70–90%	Moderate (non-recyclable, toxic byproducts)
Ullmann-type coupling[4]	Cu-based	High temperature, polar solvents	High energy use, limited substrate choice	Low to moderate	Low (harsh conditions, limited substates)
This Work: Seaweed-derived PdNPs	Ligand-free PdNPs from seaweed extract	Aqueous/ethanol solvent, mild temperature	Minimizes toxic waste, recyclable catalyst	Up to 98%	High (renewable catalyst source, mild and green)

References:

1. Remuzzi, G. (Merck Sharp and Dohme Italia S P (IT)), U.S. Patent 6,576,652, **2003**.
2. Carini, D.J.; Duncia, J.V.; Aldrich, P.E.; Chiu, A.T.; Johnson, A.L.; Pierce, M.E.; Price, W.A.; Santella III, J.B.; Wells, G.J.; Wexler, R.R.; Wong, P.C.; Yoo, S-E; Timmermans, P.B.M.W.M. Nonpeptide angiotensin II receptor antagonists: the discovery of a series of N-(biphenylylmethyl)imidazoles as potent, orally active antihypertensives. *J. Med. Chem.* **1991**, *34*, 2525-2547.

3. Buskes, M. J.; Blanco, M.-J. Impact of Cross-Coupling Reactions in Drug Discovery and Development. *Molecules* **2020**, *25*(16), 3731.
4. Surry, D. S.; Buchwald, S. L. Biaryl synthesis via Ullmann-type coupling reactions. *Chem. Sci.* **2008**, *1*(1), 13–31.
